# Commentary: The impact of bariatric and metabolic surgery on cancer development

**DOI:** 10.3389/fsurg.2022.1032084

**Published:** 2022-09-29

**Authors:** Magdalena Taube, Kajsa Sjöholm, Markku Peltonen, Lena Carlsson

**Affiliations:** ^1^ Department of Molecular and Clinical Medicine, Institute of Medicine, the Sahlgrenska Academy at University of Gothenburg, Gothenburg, Sweden; ^2^ Public Health Promotion Unit, National Institute for Health and Welfare, Helsinki, Finland; ^3^ Department of Neurobiology, Care Sciences and Society, Karolinska Institutet, Huddinge, Sweden

**Keywords:** bariatric, surgery, cancer, colorectal, incidence

**A Commentary on**
The impact of bariatric and metabolic surgery on cancer development by Lunger F, Aeschbacher P, Nett PC, Peros G. Front Surg. (2022). 9:918272. doi: 10.3389/fsurg.2022.918272

Obesity is associated with increased cancer risk and bariatric surgery -leading to substantial, sustainable weight loss -has repeatedly been found to be associated with a reduced risk. To increase the understanding of this expanding research field review articles that summarize results from different studies are needed. One such review is the recent publication from Lunger et al. (1), in which pre- and post-interventional aspects of bariatric and metabolic surgery and its potential benefit on cancer development in patients with obesity is reviewed and discussed. We read this review with great interest, but as representatives of the Swedish Obese Subjects (SOS) study group we need to point out that the statement regarding our previously published report specifically analyzing incidence of colorectal cancer is incorrect (2).

In the review article by Lunger et al*.* it is stated that our study (2) demonstrates an increased risk of colorectal cancer with bariatric surgery and also that the risk increased steadily with time following surgery. In contrast, we found no evidence that colorectal cancer is affected by bariatric surgery (hazard ratio_unadj_ with surgery = 0.79 (95% CI: 0.55–1.12; *p* = 0.183). In addition, when analyzing rectal cancer events separately- we found a decreased risk of rectal cancer with surgery (HR_unadj_ = 0.56; 95% CI: 0.32–0.99; *p* = 0.045), while the risk of colon cancer was unchanged.
